# Allergen immunotherapy combined with Notch pathway inhibitors improves HDM-induced allergic airway inflammation and inhibits ILC2 activation

**DOI:** 10.3389/fimmu.2023.1264071

**Published:** 2024-02-02

**Authors:** Yu Tong, Lei Wang, Lingya Wang, Jingjing Song, Junwen Fan, Chuqiao Lai, Jiali Bao, Cuiye Weng, Yufei Wang, Jilong Shuai, Hui Zhang, Weixi Zhang

**Affiliations:** Department of Pediatric Allergy and Immunology, The Second Hospital and Yuying Children’s Hospital of Wenzhou Medical University, Wenzhou, China

**Keywords:** asthma, ILC2s, AIT, notch signaling pathway, HDM

## Abstract

**Introduction:**

Group 2 innate lymphoid cells (ILC2s) play a crucial role in house dust mite (HDM)-induced allergic inflammation, and allergen immunotherapy (AIT) holds promise for treating the disease by reducing the frequency of ILC2s. Despite significant progress in AIT for allergic diseases, there remains a need to improve the control of allergic symptoms.

**Methods:**

We investigated the synergistic effect of the Notch signaling pathway and subcutaneous immunotherapy (SCIT) in treating allergic airway inflammation in mice and their impact on the ratio of ILC2s in lung tissues. This was achieved by establishing the HDM-induced airway allergic disorders (HAAD) model and SCIT model. Additionally, we conducted *in vitro* investigations into the effect of the Notch signaling pathway on the secretory function of activated ILC2s using fluorescence-activated cell sorting. Furthermore, we explored the coactivation of the Notch signaling pathway with SCIT *in vitro* by sorting ILC2s from the lung tissues of mice after SCIT modeling.

**Results:**

Previously, our group demonstrated that Notch signaling pathway inhibitors can reduce allergic airway inflammation in mice. Notch signaling induces lineage plasticity of mature ILC2s. In this study, we showed that AIT alleviates allergic airway inflammation and suppresses the frequency of ILC2s induced by HDM. Interestingly, AIT combined with a γ-secretase inhibitor (GSI), an inhibitor of the Notch signaling pathway, significantly inhibited the frequency of ILC2s, reduced airway inflammation, and suppressed Th2-type responses in a mouse model. Furthermore, lung ILC2s from HDM-challenged mice with or without AIT were treated with GSI *in vitro*, and we found that GSI dramatically reduced the secretion of type 2 inflammatory factors in ILC2s.

**Discussion:**

These findings suggest that Notch signaling pathway inhibitors can be used as adjuvant therapy for AIT and may hold potential treatment value in the cooperative control of allergic airway inflammation during early AIT.

## Introduction

Asthma is an airway disease characterized by airway hyperresponsiveness (AHR), airway inflammation, and increased mucus production. Overall, more than half of asthmatic adults and pediatric patients have allergic asthma (1). House dust mites (HDMs) are the most prevalent allergen among allergic asthma patients (2). Although the rate of allergic asthma decreases as the age of onset increases (3), airway damage from childhood asthma can persist into adulthood (4). Historically, allergic asthma was thought to be an inflammatory disease associated with T-helper 2 (Th2) cells (5). More recently, Group 2 innate lymphoid cells (ILC2s) have also been considered as playing an overwhelming role in the inflammatory response to allergic asthma (6, 7).

Allergen immunotherapy (AIT), which aims to prevent the development of new allergic conditions, is extensively utilized in the clinical treatment of house dust mite (HDM) allergic diseases. This therapy primarily encompasses subcutaneous immunotherapy (SCIT) and sublingual immunotherapy (SLIT) (8). Although much clinical research has demonstrated that AIT has considerable therapeutic effects (9–14), it is not effective for everyone. Therefore, attempts have been made to find new ways to enhance the effect of AIT. AIT is considered to work by reversing allergen-induced modifications in the balance between pro-inflammatory and anti-inflammatory T cells. During allergen immunotherapy (AIT), exposure to high doses of allergens can have dual effects. On one hand, it can induce epithelial cells to secrete anti-inflammatory mediators, such as interleukin (IL)-37 (15) and secretoglobin1A1 (16, 17). These mediators play a crucial role in modulating airway allergic responses by inhibiting pro-inflammatory factors. On the other hand, the presence of transforming growth factor (TGF)-β can induce the proliferation of a specific subset of regulatory T cells (Treg cells). This expansion of Treg cells further contributes to the regulation of immune responses during AIT.These cells decrease Th2 cell responses while inducing a shift to Th1 cell responses (18).

Notably, AIT has been shown to modulate ILC2s. Clinical studies have found a sustained decrease in ILC2s in the peripheral blood of allergic patients after AIT (19–21). Innate lymphoid cells (ILCs), which lack antigen-specific receptors and predominantly reside in mucosal tissues (22, 23), are a subset of ILCs. Among them, ILC2s express the transcription factor GATA-binding Factor 3 (GATA3) and play a role similar to Th2 cells by releasing type 2 cytokines such as IL-13 and IL-5 (24–26). Undeniably, ILC2s play an integral part in allergic asthma (27). When exposed to allergens, epithelial cells release epithelial cell-derived cytokines, such as IL-33, IL-25, and thymic stromal lymphopoietin (TSLP), which activate ILC2s, resulting in a type 2 immune response (28, 29).

The Notch signaling pathway, which is broadly observed in mammals and other animals, plays an integral role in the growth and evolution of lymphocytes (30). In our previous experiments, it was found that γ-secretase inhibitors (GSIs) mitigated airway inflammation by blocking the Notch signaling pathway in OVA-induced asthmatic mice (31, 32). Similarly, the Notch signaling pathway is critical for the development of ILC2s. It has been discovered that Notch signaling is necessary for the accumulation of inflammatory group 2 innate cells in the lung (33). Natural ILC2s were cultivated *in vitro* in IL-2, IL-7, and IL-25 conditions, and the activation of the Notch signaling pathway dramatically boosted the presence of ILC2s, as well as the expression of IL-13 and IL-5 (34). More crucially, precursor cells, rather than T cells, drive the formation and differentiation of ILC2s under conditions of high Notch signaling intensity (35). However, it remains elusive as to whether the Notch signaling pathway regulates the activation of ILC2s and interferes with the efficacy of AIT treatment for allergic asthma.

In this study, we investigated the effect of GSI on the function of ILC2s in the context of HDM-induced allergic airway inflammation. Our findings revealed that GSI inhibits the secretion of cytokines by ILC2s. We established a mouse model of HDM-induced airway allergic disorders (HAAD) and treated HAAD mice with SCIT, with or without GSI. Our results demonstrated that GSI significantly enhanced the effectiveness of SCIT for HAAD and suppressed the response of ILC2s.

## Materials and methods

### Mice

Wild-type BALB/c mice were obtained from Hangzhou Charles River Laboratories, China. All mice were housed in a specific pathogen-free environment, and female mice weighing 18-20 g were used for experiments. All experiments on animals were conducted in accordance with ethical protocols approved by the Laboratory Animal Ethics Committee of Wenzhou Medical University & Laboratory Animal Centre of Wenzhou Medical University.

### Mouse model of HAAD

**Figure 1:**
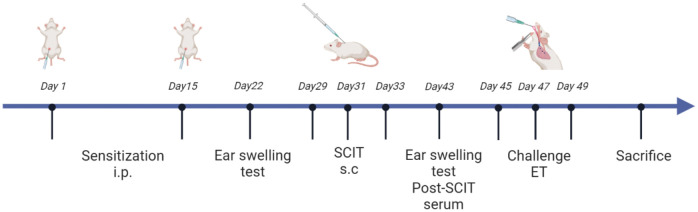
HDM-induced subcutaneous immunotherapy in asthmatic mice as a paradigm, created in BioRender.com.

All mice were randomly assigned to one of four groups: the control group, HDM group, HDM+SCIT group, and HDM+SCIT+GSI group. In this study, we established a model of HAAD and SCIT as described in **Figure 1** and **Supplementary Table 1**. Mice weighing 18-22 g received an intraperitoneal injection of 5 μg HDM (XPB91D3A25, Greer Laboratories, Lenoir, North Carolina, USA) adsorbed on 2.25 mg of aluminum hydroxide in 100 μl PBS on Days 1 and 15. The mice in the HDM+SCIT and HDM+SCIT+GSI groups were desensitized through subcutaneous injection of 250 μg HDM in 100 μl PBS on Days 29, 31, and 33 (36). The mice in the control group were injected with PBS. In the HDM+SCIT+GSI group, GSI L685, 458 (Merck, USA) (0.3 mg/kg) was given to the mice intratracheally 30 minutes (37–39) before each SCIT. Ultimately, the mice were challenged with 25 μg of HDM in 25 μl of PBS via intratracheal direct injection on Days 45, 47, and 49.

### Ear swelling test

The ear swelling test (EST) (36) was measured to assess the early phase reaction to HDM before and after SCIT on Days 22 and 32, in order to evaluate the allergic sensitization of the mice. Following anesthesia with isoflurane/oxygen, mice were intradermally injected with 10 μl PBS containing 0.5 μg HDM in the right ear and subcutaneously with 10 μl PBS in the left ear as a control. After 2 hours, the thickness of the mouse ears was measured using a digimatic micrometer (Mitutoyo, Japan), and the thickness of the right ear was subtracted from the thickness of the left ear to calculate the degree of HDM-induced swelling.

### Measure of lung function

After the final HDM challenge, mice were anesthetized via intraperitoneal injection of 1% pentobarbital sodium. Once the mice had completely ceased spontaneous respiration, they were immobilized and the trachea was surgically incised for tracheal intubation. Subsequently, the inserted tracheal tube was connected to SCIREQ Scientific Respiratory Equipment (FlexIVent, Montreal, Canada), and the intermittent ventilation mode (150 breaths/min, PEEP 2 cmH_2_O) was selected. Baseline airway resistance (Rn) was measured at the stage of saline nebulizing. Then, the mice were challenged with an increasing dose (3.125, 6.25, 12.5, 25, and 50 mg/ml) of methacholine (MCh). And airway Rn was measured at every dose to calculate the percentage of each concentration to baseline.

### BAL fluid collection

After evaluating the lung function of the mice, they were cannulated, and the lungs were washed three times with 0.8 ml of cold PBS. The supernatant was collected by centrifuging the bronchoalveolar lavage (BAL) fluid for ELISA, and the cells were resuspended in cold PBS for counting and analyzed by flow cytometry (FCM).

### Western blot

The total protein in lung tissue was isolated and quantified. Protein lysates were electrophoresed using the PAGE Gel Fast Preparation Kit (Epizyme Biotech, China) and then electrotransferred to a polyvinylidene fluoride (PVDF) membrane. The resulting blots were blocked with 5% milk and incubated overnight with the following primary antibodies: anti-Notch1 (Abcam, UK), anti-Hes-1 (Cell Signaling Technology, USA), and anti-tubulin (Affinity Biosciences, USA). Subsequently, goat anti-rabbit IgG-HRP (Affinity, China) as the secondary antibody was incubated at room temperature for 30 minutes.

### Quantitative real-time polymerase chain reaction

TRIzol (GlpBio, USA) was employed to extract total RNA from lung tissue. Next, cDNA was synthesized by reverse transcription using qRT Master Mix (TOROIVD, China) according to the manufacturer’s instructions. To examine gene mRNA expression, qPCR (Takara, Japan) was performed with SYBR Green PCR Master Mix. **Supplementary Table 3** shows the primer sequences that were employed.

### Cell isolation from lung tissues

Mouse lung tissues were removed and cleaned in PBS to remove residual blood after right heart lavage with cold PBS under aseptic conditions. Fresh tissues were diced and digested in RPMI1640 medium containing 1 mg/ml Collagenase Type IV (Worthington, USA) and 0.2 mg/ml DNase I (Sigma, UAS) in a 37°C cell incubator for 1 h. Digested tissues were ground and filtered on a 70 μm filter and then further isolated and purified with Ficoll gradient (TBDscience, China). Naturally, the isolated cells were lysed by red blood cell lysis buffer to obtain a single-cell suspension of lung tissues for the following experiments.

### Flow cytometric analysis and ILC2 sorting

Before staining with fluorochrome-conjugated antibodies, which are listed in **Supplementary Table 2**, single-cell suspensions were blocked with anti-Fc receptor antibodies (anti-mouse CD16/32). For the flow cytometric fluorescence sorting (FACS), a cell viability dye (7-AAD, BD Bioscience, USA) was used to exclude dead cells. Afterward, fluorochrome-conjugated antibodies were used for surface dyeing (lineage antibodies, anti-CD45, anti-ST2, anti-CD127). We defined the population of cells expressing Lin^-^CD45^+^CD127^+^ST2^+^ as ILC2s. The lineage antibodies we used included anti-CD3e, anti-CD11b, anti-CD45R/B220, anti-TER-119, anti-Ly-6G, and anti-Ly-6C, and then a MoFlo Astrios EQ (Beckman Coulter, USA) was employed for sorting. The purity of isolated ILC2s was up to 96.2%. For intracellular transcription factor staining, sorted ILC2s were handled with the Foxp3/Transcription Factor Staining Buffer Set (eBioscience, USA) according to instructions of the manufacturer after *in vitro* culture and stained with fluorochrome-conjugated antibodies anti-GATA3 antibodies at 4°C for 1.5 h.

### *In vitro* culture of ILC2s

To investigate the function of ILC2s *in vitro*, lung ILC2s were isolated from the HDM group and HDM+SCIT group, cultured on 96-well plates at a density of 5×10^3^ per well, and processed with GSI (20 μM/ml) or PBS in the presence of mouse IL-2 (10 ng/ml, PeproTech, USA) and IL-7 (10 ng/ml, PeproTech, USA). The intracellular transcription factor GATA3 of ILC2s was analyzed by FCM after stimulation for 16 h, while the cytokines in the culture supernatant were detected by ELISA after stimulation for 48 h.

### Cytokine measurements

The BAL fluid and serum from mice, as well as the supernatant from cultured ILC2s, were collected and stored at -80°C. Levels of the cytokines IL-4, IL-5, IL-13, and IgE were determined by ELISA as described by the manufacturer (Thermo Fisher, USA). To detect HDM-sIgE, we coated HDMs (10 μg/plate) on 96-well plates and incubated them overnight at 4°C. After washing with PBST, the coated 96-well plates were blocked with assay diluent buffer for 2 h at room temperature. Then, a 1:80 dilution of serum was added to the plate and incubated at room temperature for 2 h. Subsequently, after washing with PBST, the detection antibody (1:1000 diluted, Biotin Rat Anti-Mouse, BD Pharmingen, USA) was added to the plate and incubated at room temperature for 1 h. Next, 100 μl 1× streptavidin-horseradish peroxidase (HRP) was added to the 96-well plate and incubated for half an hour at room temperature. At last, 100 μl TMB was added to the plate and incubated for 15 min and then the reaction was stopped by adding stop solution. The absorbance of the plate was detected at 450 nm using an enzyme marker. The concentration of total IgE ranged from 4 to 250 ng/ml, with a detection range of 4-500 pg/ml for IL-4, IL-5, and IL-13.

### Histopathology

Fresh lung tissue in 4% paraformaldehyde solution fixation for 48 h, followed by paraffin embedding. After tissue sectioning, hematoxylin-eosin (H&E) staining, Masson staining, and Alcian Blue Periodic acid Schiff (AB-PAS) staining were followed. Airway inflammation scores were referenced from our previous report (31, 37).

### Data analysis

All data are expressed as the mean ± SEM and statistical significance was determined by one-way ANOVA or Student’s two-tailed t-test. GraphPad Prism 8.0 software was used for statistical analysis, and FlowJo 10.6.2 software was employed for FCM analysis. *P*<0.05 was recognized as a significant difference.

## Results

### GSI inhibits GATA3 expression and cytokine secretion by ILC2s *in vitro*

**Figure 2:**
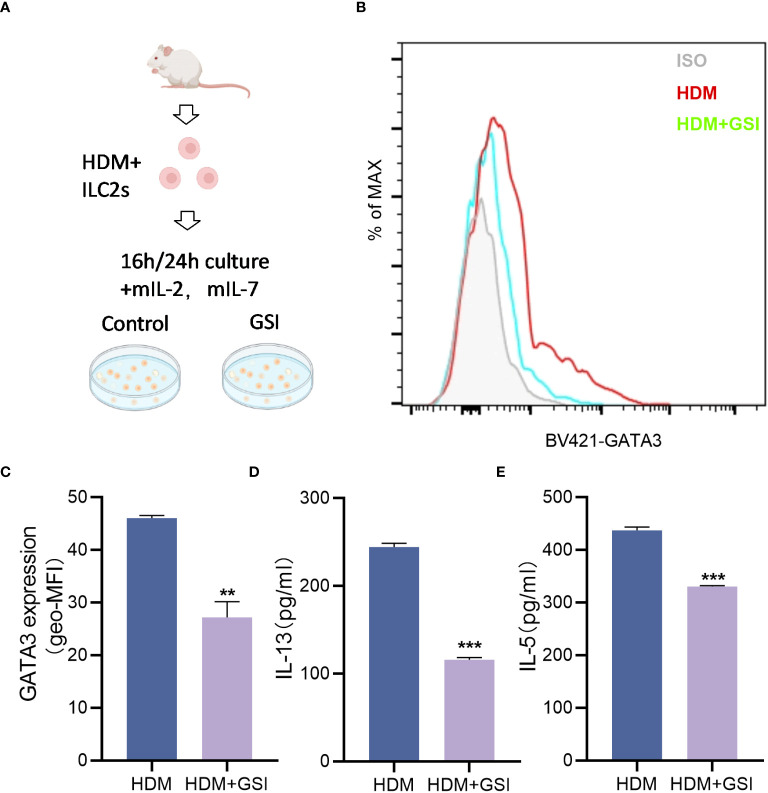
GSI inhibits intracellular GATA3 expression and type 2 cytokine generation in ILC2s *in vitro*. **(A)** Diagram showing HDM-induced asthmatic mouse lung tissue extracted and cultivated *in vitro* using FACS for ILC2s. **(B, C)** The mean fluorescence intensity (MFI) of GATA3 within ILC2s was measured by FCM after 16 h incubation of ILC2s in the presence of PBS/GSI. **(D, E)** ILC2s were cultured in the presence of PBS/GSI for 24 h and then the expression levels of IL-5 and IL-13 were measured by ELISA. The data represent three independent experiments, and mean ± SEMs are shown. Student’s two-tailed t-test was used. ^**^ *P <*0.01, ^***^ *P <*0.001 compared with control group.

To investigate the potential impact of GSI on ILC2s function, we primarily evaluated its effect on the production of type 2 cytokines by HDM-activated ILC2s. To promote the expansion of ILC2s, BALB/c mice were pretreated with HDM, and lung ILC2s were isolated as Lin^-^CD45^+^ST2^+^CD127^+^ cells (**Figure 2A**) using the sorting gating strategy (S3). We treated sorted lung ILC2s with GSI and observed a notable decrease in the levels of IL-13 and IL-5 in the supernatant of cultured ILC2s, as well as a reduction inintracellular GATA3 expression levels in ILC2s (**Figures 2B–E**).

### Notch expression is elevated in the lung tissues of the HAAD mouse mice model

**Figure 3:**
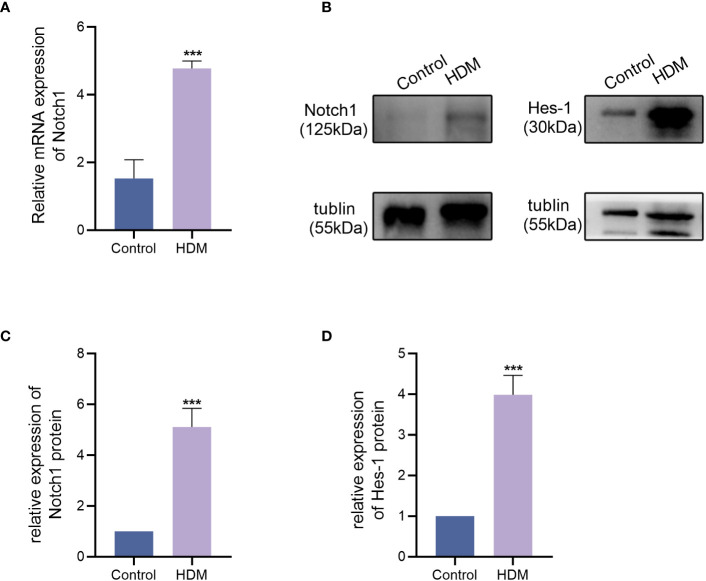
Notch1 and Hes-1 protein expression levels, as well as Notch1 mRNA expression levels, were measured. **(A)** mRNA expression level of Notch1. **(B–D)** Protein expression level of Notch1 and Hes-1. These data represent at least three independent experiments, and mean ± SEMs were shown. Student’s two-tailed t-test was used. ^***^ *P <*0.001 compared with the control group.

To examine the expression of Notch in the HAAD model, lung tissues were harvested and assessed using western blotting and qPCR. As shown in **Figure 3**, the mRNA expression of Notch1 was significantly higher in the lung tissue of the HDM group compared to the control group. Interestingly, the protein expression of Notch1 and its downstream target Hes-1 was dramatically elevated in the lung tissue of HDM mice compared to the control group. These findings indicate that HDM induces an increase in Notch signaling expression in the lung tissue of asthmatic mice.

### GSI combined with SCIT further alleviates AHR in HAAD mice

**Figure 4:**
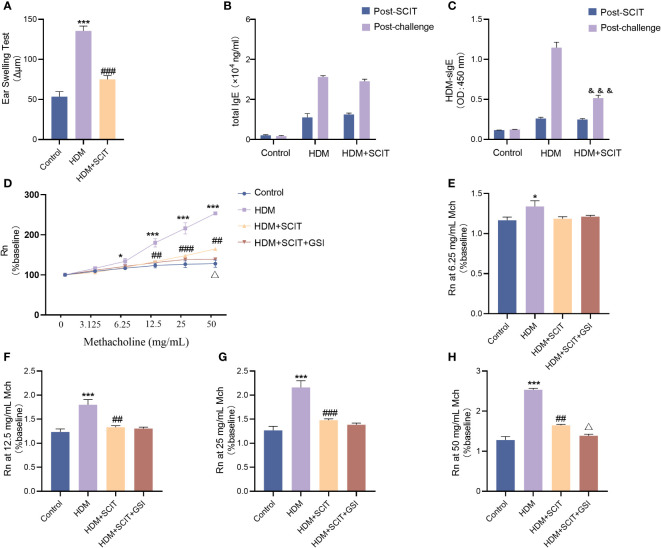
GSI combined with SCIT attenuates AHR in mice of HAAD. Experiment diagram of animal model and changes in airway resistance of each group. **(A)** EST reaction early allergic reaction. **(B)** Change in total IgE before and after the challenge. **(C)** Change in HDM-sIgE before and after the challenge. **(D–H)** Comparison of airway resistance in each group of mice at different methacholine concentrations. The data represent at least three independent experiments, mean±SEMs were shown. One-way ANOVA test was used for **(A, D–H)**. *P <0.05, ***P <0.001 compared with control group. ^##^ *P* <0.01, ^###^ *P* <0.001, compared with HDM group. ^△^ *P* <0.05, compared with HDM+SCIT group. Two-way ANOVA test was used for **(B, C)**. ^&&&^ *P* <0.01, compared to HDM group post challenge.

To begin with, we evaluated the extent of ear swelling in mice before and after SCIT treatment (**Figure 4A**). We observed that ear swelling was significantly more severe in the asthma group of mice compared to the control group (*P*<0.001), but it was reduced after SCIT treatment compared to the asthma group (*P*<0.001). This indicates that SCIT therapy suppresses early allergic reactions in mice. Subsequently, we measured total serum IgE and HDM-specific IgE (HDM-sIgE) levels in mice post-SCIT and challenge (**Figures 4B, C**). The total serum IgE levels in the SCIT group post-SCIT and challenge were not statistically significant compared to the asthma group. However, serum HDM-sIgE levels in the SCIT group were significantly lower post-challenge than those in the asthma group (*P*<0.001). These results suggest a successful SCIT model. Airway resistance (Rn) was evaluated after the last challenge. The results (**Figures 4D–H**) showed that airway resistance was higher in the HDM group of mice compared to the control group (*P*<0.5), while SCIT significantly reduced Rn (*P*<0.5). Interestingly, the decrease in airway resistance was more pronounced in the HDM+SCIT+GSI group mice compared to the HDM+SCIT group(*P*<0.5). These observations suggest that GSI in combination with SCIT can attenuate HDM-induced AHR and early allergic reactions in mice.

### GSI together with SCIT decreases airway inflammation, collagen deposition, and goblet cell hyperplasia

**Figure 5:**
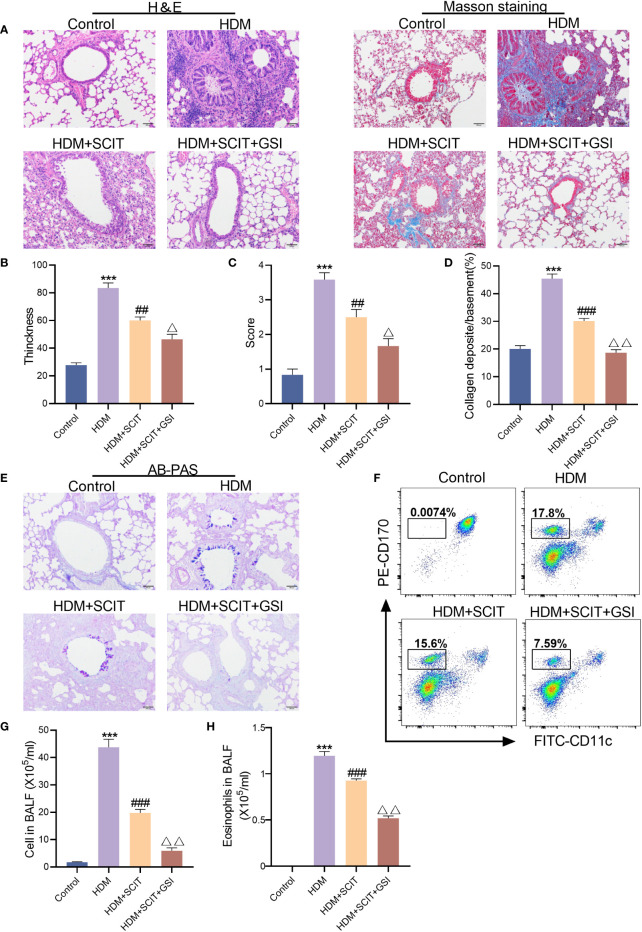
GSI and SCIT together reduce airway inflammation and inhibit airway collagen fibrosis and mucus secretion. **(A)** H&E staining and Masson staining of lung pathology sections (bars,100 μm). **(B)** Thickness of tracheal wall. **(C)** Semi-quantitative analysis to assess lung tissue inflammation. **(D)** Quantitative analysis of the percentage of peritracheal collagen deposition. **(E)** AB-PAS staining of lung pathology sections (bars,100 μm). **(F)** Eosinophil flow gating strategy. **(G)** Tatal cell in BAL fluid. **(H)** Eosinophils in BAL fluid. The data represent at least three independent experiments, mean±SEMs were shown. One-way ANOVA test was used. ***P <0.001 compared with control group. ^##^ *P* <0.01, ^###^ *P* <0.01, compared with HDM group. ^△^ *P* <0.05, ^△△^ *P* <0.01, compared with HDM+SCIT group.

The lung histopathological sections were stained with H&E(**Figures 5A–C**). As expected, bronchial wall thickness decreased after SCIT and inflammatory cell infiltration in lung tissue was alleviated (*P*<0.5). The total cell count and eosinophil count of BAL fluid were significantly reduced (**Figures 5F–H**). More significantly, inflammatory cell infiltration in the lungs of mice in the HDM+SCIT+GSI group was considerably lower than in the HDM+SCIT group (**Figures 5A–C**). Similarly, the HDM+SCIT+GSI group showed the most prominent decrease in BALF total cell count and eosinophil count compared to the HDM+SCIT group (**Figures 5F–H**).

Afterward, to further investigate the effect of GSI combined with SCIT on asthma, lung tissues of mice were examined using Masson staining (**Figures 5A, D**) and AB-PAS staining (**Figure 5E**). We found that SCIT dramatically reduced peritracheal collagen deposition (*P*<0.5) and decreased airway mucus secretion. Unexpectedly, the percentage of peritracheal collagen deposition in mice in the HDM+SCIT+GSI group (*P*<0.5) decreased obviously compared with that in mice in the HDM+SCIT group, and airway mucus secretion was also reduced. Overall, these results indicate that GSI combined with SCIT can synergistically alleviate airway inflammation and inhibit airway collagen fibrosis and mucus secretion.

### GSI decreases type 2 inflammation on the foundation of SCIT

**Figure 6:**
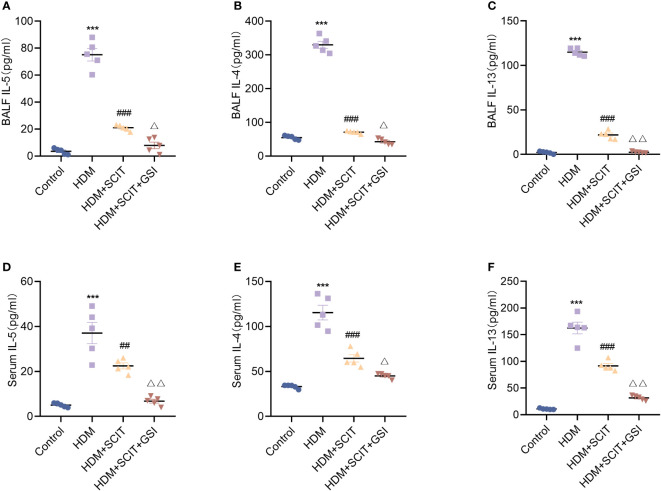
GSI further suppresses Th2-type inflammatory responses based on SCIT. **(A–C)** Assay of IL-4, IL-5 and IL-13 levels by ELISA in BAL fluid. **(D–F)** Measurement of IL-4, IL-5 and IL-13 levels by ELISA in serum. The data represent at least three independent experiments, mean±SEMs were shown. One-way ANOVA test was used. ***P <0.001 compared with control group. ^##^ *P* <0.01, ^###^ *P* <0.001, compared with HDM group. ^△^ *P* <0.05, ^△△^ *P* <0.01, compared with HDM+SCIT group.

Asthma is closely correlated with the type 2 cytokines interleukin (IL)-4, IL-5, and IL-13 (40). Therefore, we measured IL-4, IL-5, and IL-13 levels in serum and BALF using ELISA. The results (**Figure 6**) showed that IL-4, IL-5, and IL-13 levels were substantially increased in the HDM group compared to the control group (*P*<0.5), while the HDM+SCIT group (*P*<0.5) showed a significant decrease compared to the HDM group. IL-4, IL-5, and IL-13 levels were further reduced by combining SCIT with GSI treatment (*P*<0.5). These results indicate that combining SCIT and GSI can effectively reduce inflammatory cytokines in BAL fluid and serum.

### SCIT combined with GSI inhibited the activation of ILC2s

**Figure 7:**
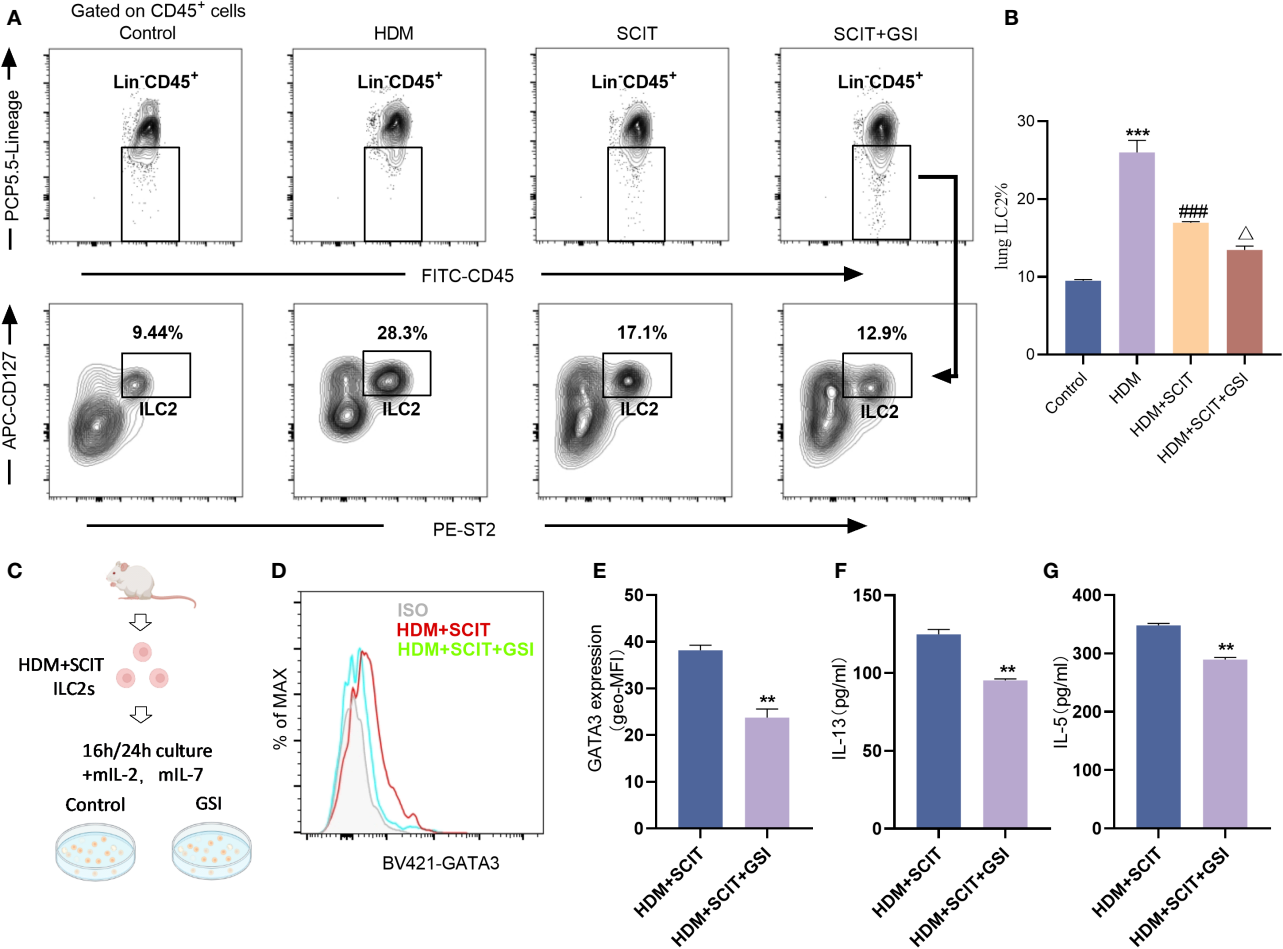
GSI combined with SCIT treatment inhibits the activation of ILC2s *in vivo* or *in vitro*. **(A)** Flow cytometry strategy for gating of ILC2s [Lin^-^CD45^+^CD127^+^ST2^+^ (41, 42)] in lung tissue. The detailed gating strategy diagram for ILC2s is shown in the **Supplementary Material Figure S2**. **(B)** Frequency of ILC2s in mouse lung tissue. The data represent at least three independent experiments, and mean ± SEMs were shown. **(C)** Schematic diagram of *in vitro* culture of ILC2s sorted from mice lung tissue after HDM challenge and SCIT treatment. **(D, E)** The mean fluorescence intensity (MFI) of GATA3 within ILC2s was measured by FCM after 16 h incubation of ILC2s in the presence of PBS/GSI. **(F, G)** ILC2s were cultured in the presence of PBS/GSI for 24 h and then the expression levels of IL-5 and IL-13 were measured by ELISA. A one-way ANOVA test was used for **(B)**. ^***^ *P <*0.001 compared with the control group. ^###^ *P <*0.001, compared with the HDM group. ^△^ *P <*0.05, compared with the HDM+SCIT group. Student’s two-tailed t-test was used for **(E–G)**. ^**^ *P <*0.01, ^***^ *P <*0.001 compared with control group.

Next, we investigated the effect of SCIT in combination with Notch signaling pathway inhibitors on ILC2s. We first evaluated the ratio of lung ILC2s in the HAAD and SCIT models (**Figures 7A, B**). Using FCM, we found that the percentage of ILC2s decreased after SCIT compared to the HDM group (*P*<0.5). When SCIT and GSI were combined (*P*<0.5), the ILC2s ratio decreased significantly compared to SCIT alone. Furthermore, ILC2s were extracted by FACS from lung tissue of mice in the HDM+SCIT group and cultured *in vitro* (**Figures 7C–G**). It was discovered that inhibiting the Notch signaling pathway decreased the MFI of intracellular GATA3 in ILC2s and reduced IL-13 and IL-5 production. Overall, SCIT and GSI treatments have the potential to synergistically suppress the activation of ILC2s in mice with HAAD.

## Discussion

In this study, we found that GSI was able to inhibit the activation of ILC2s *in vitro* and further reduce the production of IL-5 and IL-13 by ILC2s after SCIT. Additionally, we observed that SCIT therapy reduces airway inflammation in an HDM-induced immunotherapy model for asthma. Interestingly, when combined with SCIT treatment, GSI can further suppress airway inflammation and decrease the generation of inflammatory factors by ILC2s. This study demonstrates that GSI improves the efficacy of SCIT in the treatment of HDM-induced asthma and reduces ILC2s activation.

Allergen combination strategies have introduced novel concepts for the treatment of allergic disorders (43). In this study, we confirmed that HAAD mice with high levels of Notch signaling and activated ILC2s can be effectively treated with a combination of GSI and SCIT. We observed that this combination therapy alleviated HDM-induced airway inflammation and airway hyperresponsiveness in mice. Previous clinical studies have shown that combining anti-IgE monoclonal antibody (omalizumab) with AIT reduced allergy symptoms but did not have a long-term tolerance effect (44, 45). Clinical phase 2a trials have also demonstrated the potential of anti-IL-33 biologics to desensitize peanut-allergic patients (46). However, monoclonal antibodies are expensive and may not be suitable for everyone (47, 48). In contrast, our study provides a new and feasible approach to allergen combination therapy, which could be a valuable addition to adjuvant therapy for AIT.

A study suggests that AIT may targetthe prostaglandin EP3 receptor, leading to a reduction in IL-13^+^ILC2s (49). Several studies have also reported that the Notch signaling pathway plays a role in regulating ILC2s and type 2 inflammation (33, 34). In this study we investigated the Notch signaling pathway in lung ILC2s. Inhibiting the Notch signaling pathway effectively blocked the activation of ILC2s induced by HDM, as evidenced by decreased GATA3 expression and reduced production of IL-13 and IL-5. GATA3 is a central regulator of ILC2s (50). Furthermore, GSI decreased the expression of GATA3 and the secretion of type 2 inflammatory factors in ILC2s isolated from HDM-challenged mice receiving SCIT. These findings suggest that GSI may enhance the efficacy of SCIT by modulating the function of ILC2s.

Overall, SCIT and GSI treatments have the potential to synergistically suppress the activation of ILC2s in HDM-induced mice. In a previous study using a sensitized mouse model, we demonstrated that the Notch signaling pathway regulates the proliferation and activation of CD4^+^ T lymphocytes (32). Blocking this pathway with an anti-Dll4 antibody reduced allergic airway inflammation in mice (32). Our clinical trial revealed a correlation between Th17/Treg dysregulation and increased Notch expression in the peripheral blood of children with allergic asthma (51), highlighting the importance of the Notch signaling pathway in asthma pathogenesis. Furthermore, we found that GSI treatment alleviated allergic airway inflammation in mouse model (32) and reduced airway hyperresponsiveness in obese mice (37). These findings suggest that GSI could be a potential therapeutic strategy in allergic asthma. In our current study, we showed that combining GSI with SCIT not only reduced airway inflammation and AHR but also decreased the frequency of ILC2s in lung tissue *in vivo* experiments. Additionally, inhibiting the Notch signaling pathway *in vitro* significantly reduced the secretion of type 2 inflammatory factors by ILC2s following SCIT treatment. These results highlight the potential of combining GSI with SCIT as a novel approach to allergen combination therapy, offering new insights for the clinical treatment of asthma.

However, there are still several unresolved issues. The use of HDM to induction of allergic airway inflammation in our study brings us closer to a clinically relevant model compared to previous OVA-induced asthma models. Moreover, the AIT model established in this experiment better simulates the effects observed in humans. However, recent research has proposed the use of low-dose allergens combined with adjuvants as an AIT strategy (52), which provides us with a new idea for model optimization. Additionally, one study found that immunotherapy promoted the production of IL-10-expressing ILC2s *in vivo* (53), which may be related to the establishment of immune tolerance in the late stages of AIT. Therefore, further investigation is needed to understand the changes in ILC2s at each stage of AIT treatment. Third, the Notch signaling pathway has been found to contribute to the establishment of sustained unresponsiveness (SU) induced by oral immunotherapy in food allergy immunotherapy (54). This may be because inhibition of the Notch signaling pathway in the early stages of AIT reduces the production of activated ILC2s, which secrete type 2 inflammatory factors. However, during immune tolerance, inhibition of the Notch signaling pathway further limits the production of immunosuppressive cells in the bone marrow, leading to different outcomes. In addition, it has been found that immunotherapy inhibits the secretion of Th2 and Th9 cells as well as TGF-β, which decreases the induction of Th2 and Th9 cells and increases Treg cells. Interestingly, neutralizing TGF-β does not affect Treg cells, leading to immune dysregulation (55). Our team’s clinical studies have found that immunotherapy suppresses inflammation by increasing Treg and reversing the Th17/Treg ratio (56). However, it remains to be investigated whether GSI combined with AIT treatment affects these cells, as well as TGF-β.

## Conclusion

In conclusion, our study revealed the cooperative effect of GSI in the treatment of asthma by SCIT, providing a new idea for the synergistic effect of GSI on the treatment of asthma by SCIT.

## Data availability statement

The raw data supporting the conclusions of this article will be made available by the authors, without undue reservation.

## Ethics statement

The animal study was approved by Laboratory Animal Ethics Committee of Wenzhou Medical University & Laboratory Animal Centre of Wenzhou Medical University. The study was conducted in accordance with the local legislation and institutional requirements.

## Author contributions

Conception: HZ, and LW, LYW. Methodology: YT and LYW. Experiments: YT, LW, LYW, JJS, JF, CL, JB, CW YW, and JLS. Data analysis: YT, LW. Statistics: LYW, JJS and JF. Writing of the manuscript: YT and HZ. Study supervision, project administration, and funding acquisition: HZ and WZ. All authors contributed to the article and approved the submitted version.
