# Supplementary material for: Allergen immunotherapy combined with Notch pathway inhibitors improves HDM-induced allergic airway inflammation and inhibits ILC2 activation
Source: Front Immunol. 2024 Feb 2;14:1264071. doi: 10.3389/fimmu.2023.1264071 (PMC10869474; doi:10.3389/fimmu.2023.1264071)
Supplement: Supplementary file 1 [file DataSheet_1.docx]

Supplementary Material

Allergen immunotherapy combined with Notch pathway inhibitors improves HDM-induced allergic airway inflammation and inhibits ILC2 activation

Yu Tong^1†^, Lei Wang^1†^, Lingya Wang^1^, Jingjing Song^1^, Junwen Fan^1^, Chuqiao Lai^1^, Jiali Bao^1^, Cuiye Weng^1^, Yufei Wang^1^, Jilong Shuai^1^, Hui Zhang^1*^, Weixi Zhang^1*^

*** Correspondence:**

Weixi Zhang^*^ Hui Zhang^*^

[zhangweixi112@163.com](mailto:zhangweixi112@163.com) zhh855@126.com

# Supplementary Tables

Table 1 Drug dose for each group of mice

| Group | Sensitization | SCIT | Challenge |
| --- | --- | --- | --- |
| Control | PBS | PBS | PBS |
| HDM | 5 μg HDM/Alum | PBS | 25 μg HDM |
| HDM+SCIT | 5 μg HDM/Alum | 250 μg HDM | 25 μg HDM |
| HDM+SCIT+GSI | 5 μg HDM/Alum | GSI+250 μg HDM | 25 μg HDM |

Table 2 Antibodies used in this study

| Antibody | Clone | Manufacturer |
| --- | --- | --- |
| anti-CD3e | 145-2C11 | BD Bioscience |
| anti-CD11b | M1/70 | BD Bioscience |
| anti-CD45R/B220 | RA3-6B2 | BD Bioscience |
| anti-Ly-76 | TER-119 | BD Bioscience |
| anti-Ly-6G | RB6-8C5 | BD Bioscience |
| anti-Ly-6C | RB6-8C5 | BD Bioscience |
| 7-AAD | — | BD Bioscience |
| BB515 Rat Anti-Mouse CD45 | 30-F11 | BD Bioscience |
| PE Rat Anti-Mouse IL-33R | U29-93 | BD Bioscience |
| APC Rat Anti-Mouse CD127 | SB/199 | BD Bioscience |
| Purified Rat Anti-Mouse CD16/32 | 2.4G2 | BD Bioscience |
| Alexa Fluor 647 Rat IgG2a, κ Isotype Control | R35-95 | BD Bioscience |
| PE Mouse IgG1, κ Isotype Control | MOPC-21 | BD Bioscience |
| FITC anti-mouse CD11c Antibody | N418 | BioLegend |
| PerCP/Cyanine5.5 anti-mouse CD45 Antibody | 30-F11 | BioLegend |
| PE anti-mouse CD170 (Siglec-F) Antibody | S17007L | BioLegend |
| Anti-Notch1 antibody | — | Abcam |
| Anti-Hes-1 antibody | — | Cell signaling technology |
| Goat Anti-Rabbit IgG (H+L) HRP | — | Affinity Biosciences |

Table 3 Primers used for Real-time PCR

| Gene name | Forward primer (5’-3’) | Reverse primer (5’-3’) |
| --- | --- | --- |
| 18S | AGTCCCTGCCCTTTGTACACA | CGTTCCGAGGGCCTCACT |
| Notch1 | CCAGCAAGAAGAAGCGGAGAGAG | TTGTCGTCCATCAGAGCACCATC |

**Supplementary figures**


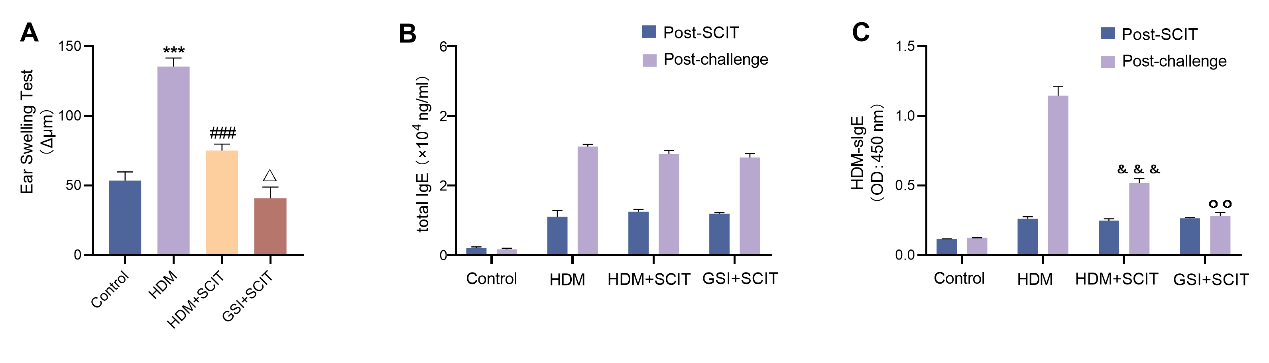


S1.The results of (A) EST, (B) total IgE, and (C) HDM-sIgE. **P* <0.05, ***P* <0.01, ****P* <0.001 compared with control group. #*P* <0.05, ##*P* <0.01, ### *P* <0.001, compared with HDM group. △*P* <0.05, compared with HDM+SCIT group, ＆＆＆*P* <0.01, compared to HDM group post-challenge. ⭕⭕ *P* <0.01, compared to HDM+SCIT group post-challenge.


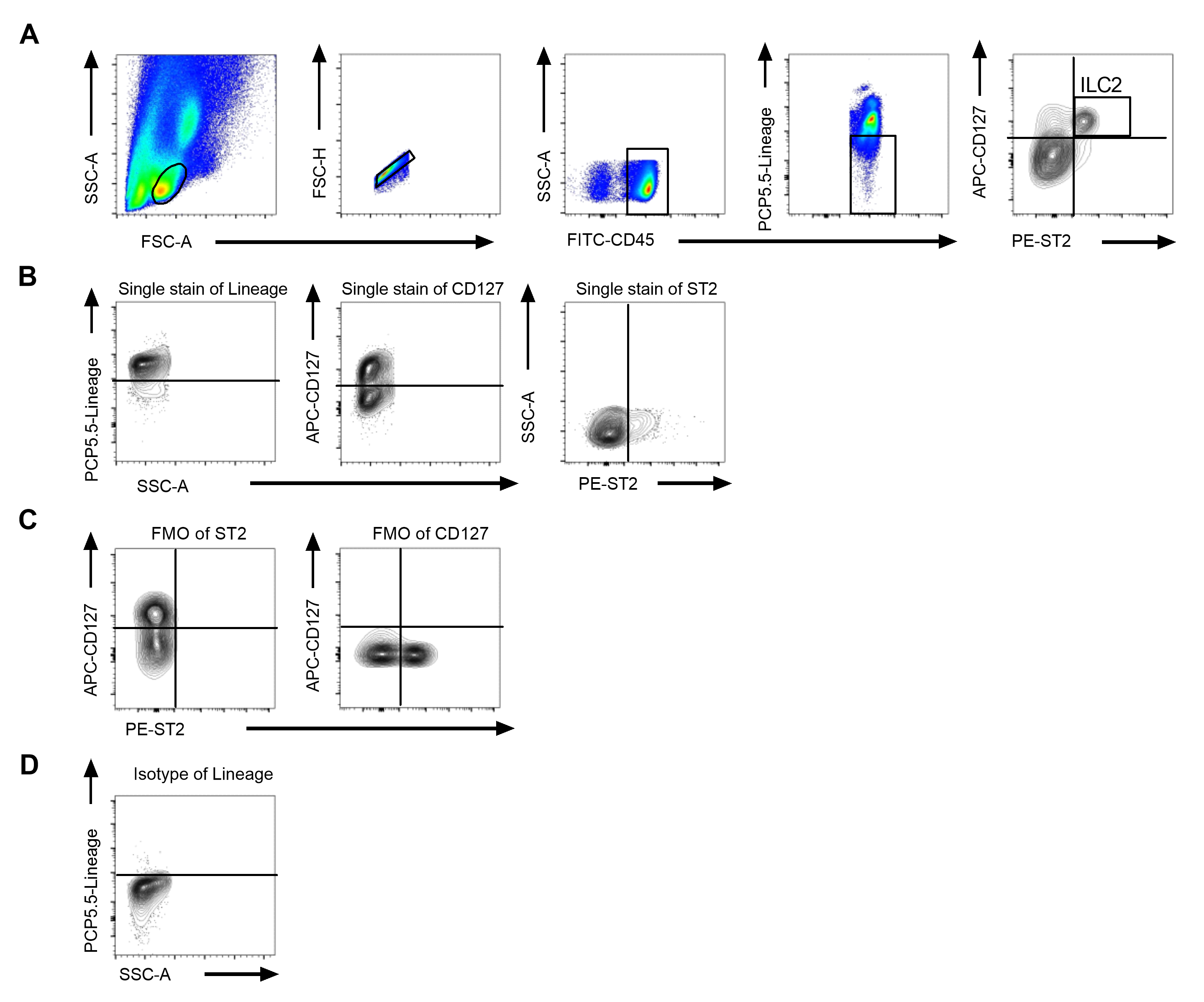


S2.Gating strategies for ILC2s. (A) Detailed gating strategy diagram for ILC2s. (B) Single color staining for Lineage, CD127, and ST2. (C) FMO-control of CD127 and ST2. (D) Isotype control for Lineage


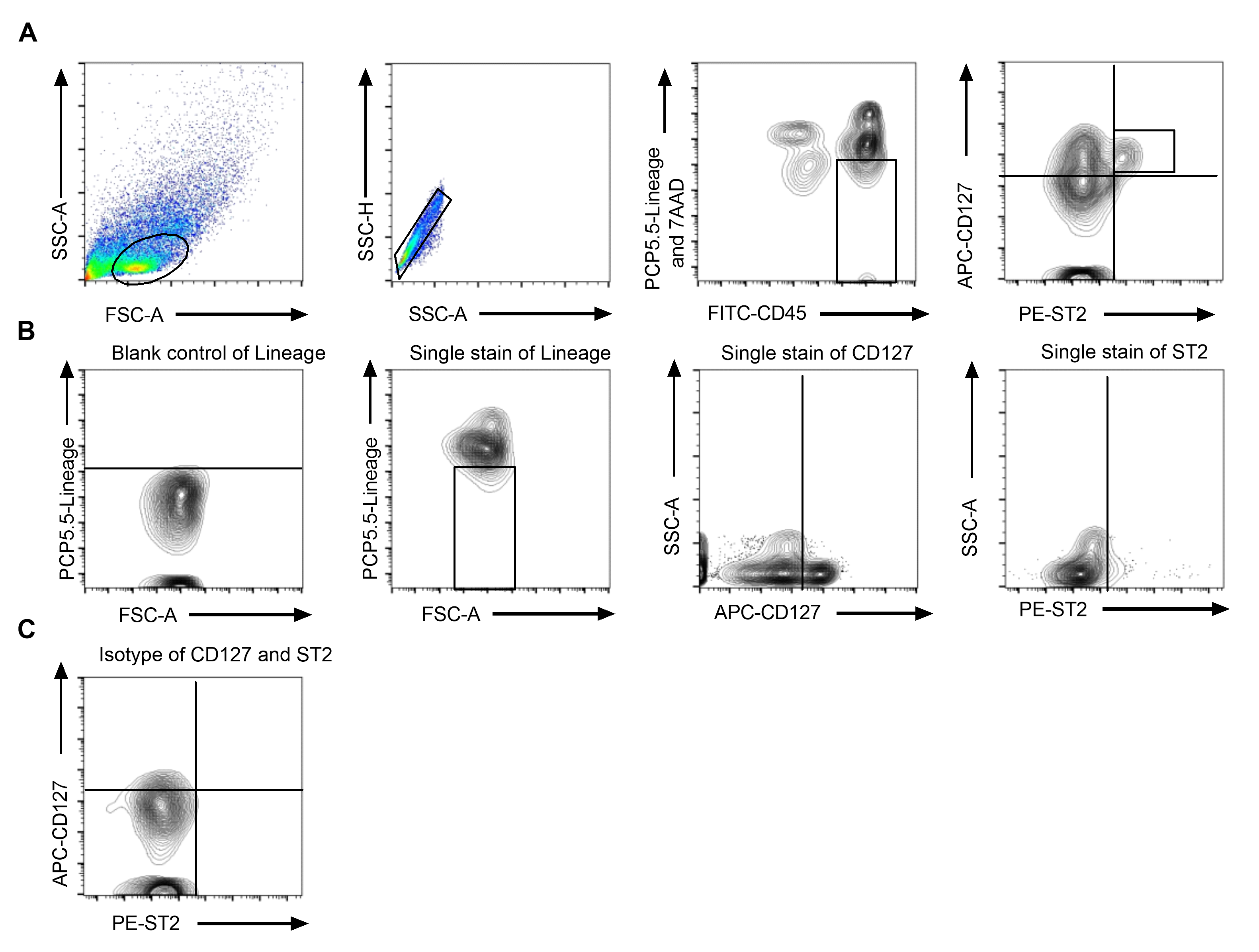


S3. ILC2s Sorting Gating Strategy. (A) Detailed sorting gating strategy diagram for ILC2s. (B) Blank control of lineage, and single-color staining for Lineage with 7AAD, CD127, and ST2. (C) FMO-control of CD127 and ST2.


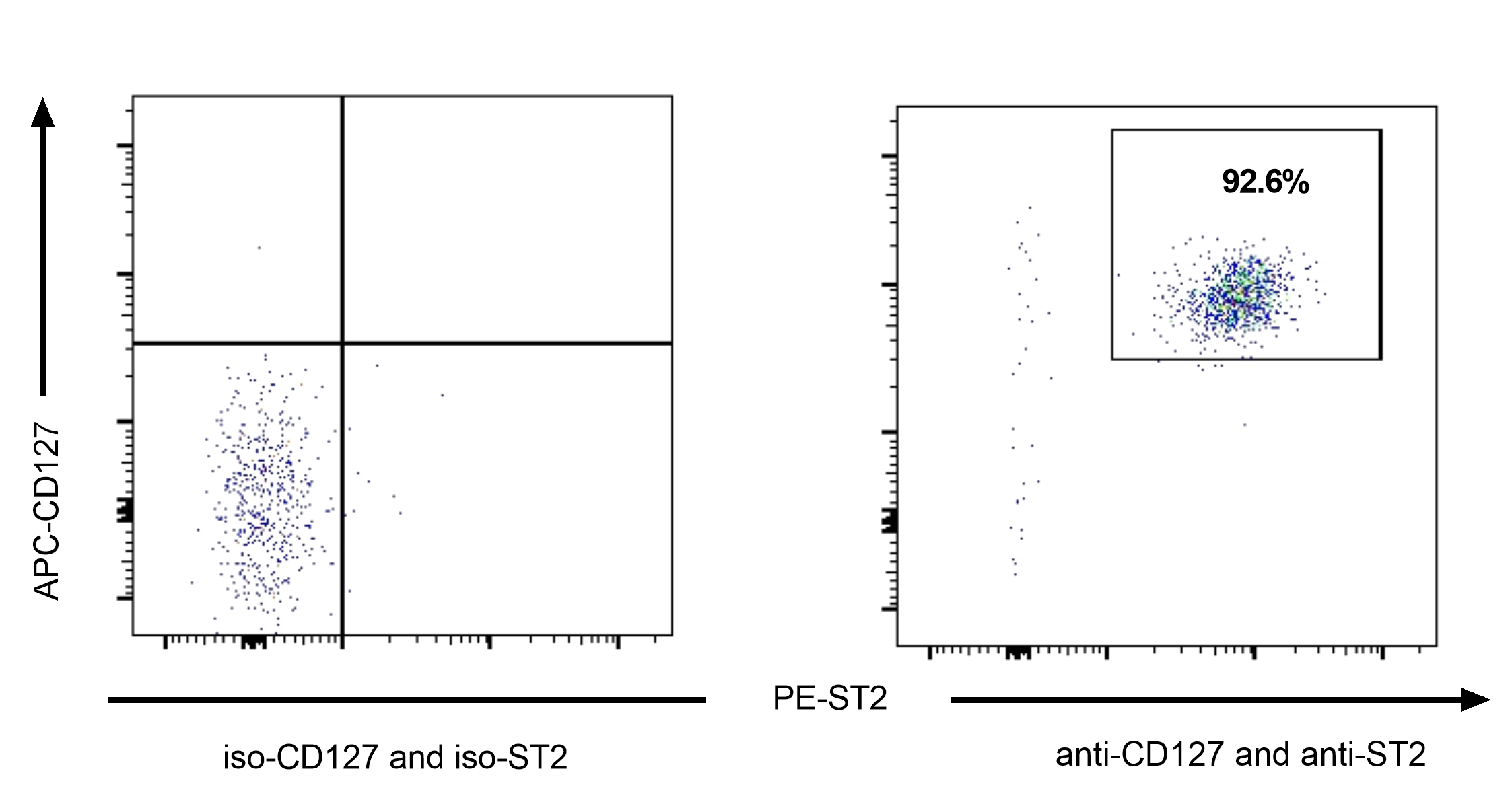


S4.The purity of ILC2s. (A) Isotype controls for CD127 and ST2, (B) Purity of ILC2s.


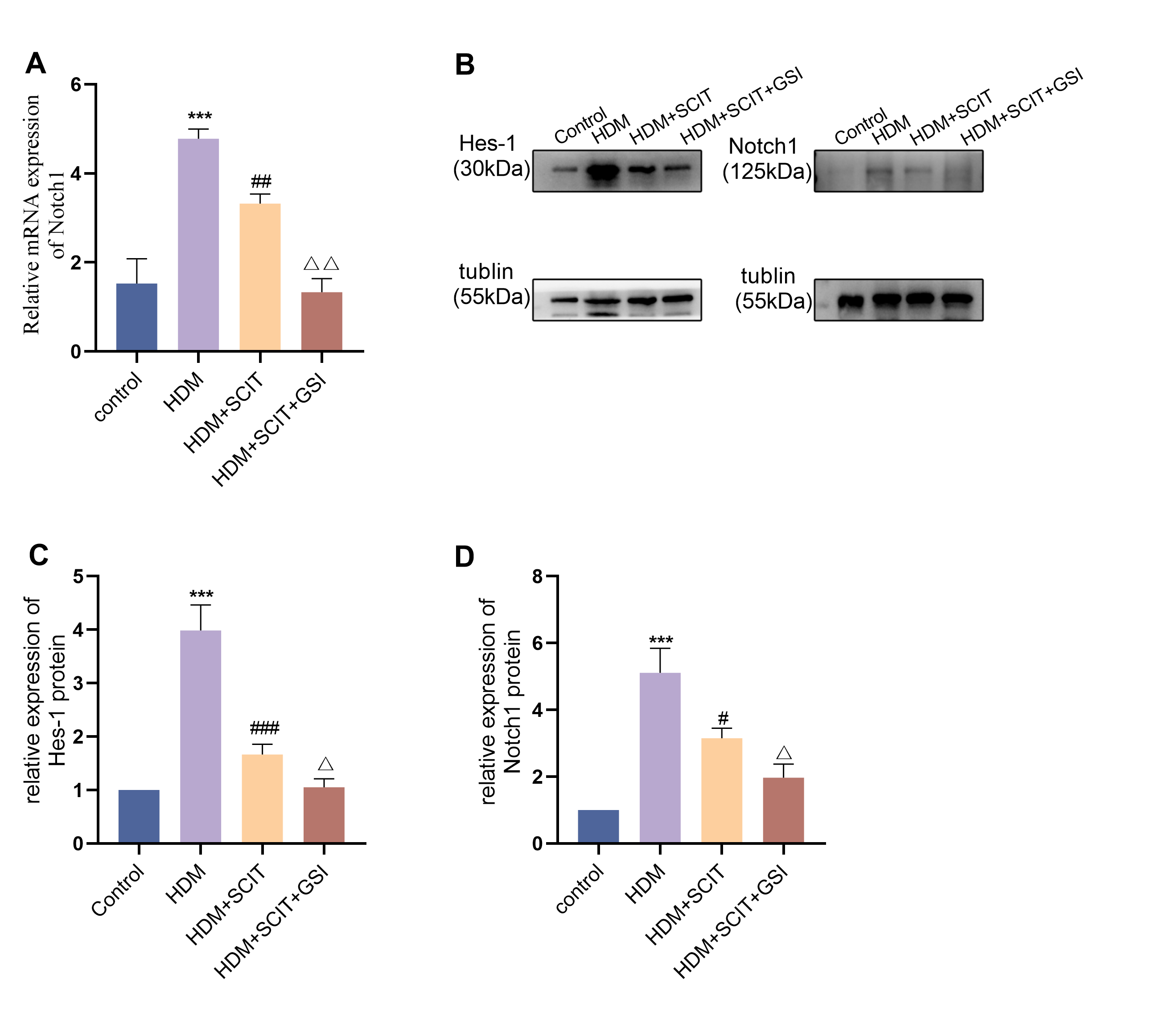


S5.Notch1 and Hes-1 protein expression levels, as well as Notch1 mRNA expression levels, were measured. (A) mRNA expression level of Notch1. (B-D) Protein expression level of Notch1 and Hes-1. These data represent at least three independent experiments, and mean±SEMs were shown. A one-way ANOVA test was used. ^***^*P* <0.001 compared with the control group. ^#^*P* <0.05, ^##^*P* <0.01, ^###^ *P* <0.001, compared with HDM group. ^△^*P* <0.05, compared with the HDM+SCIT group.


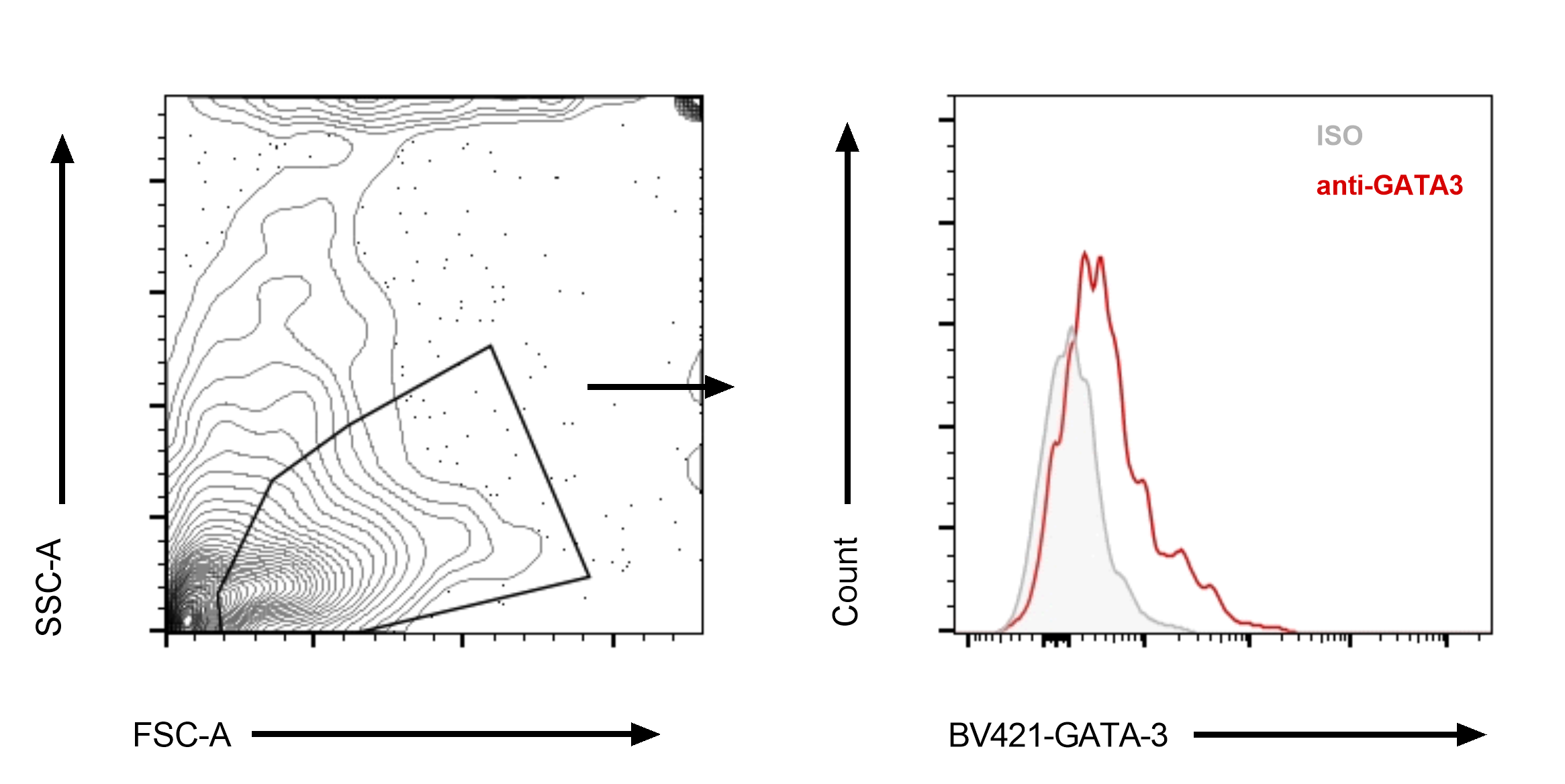


S6.Gating strategy for GATA3.


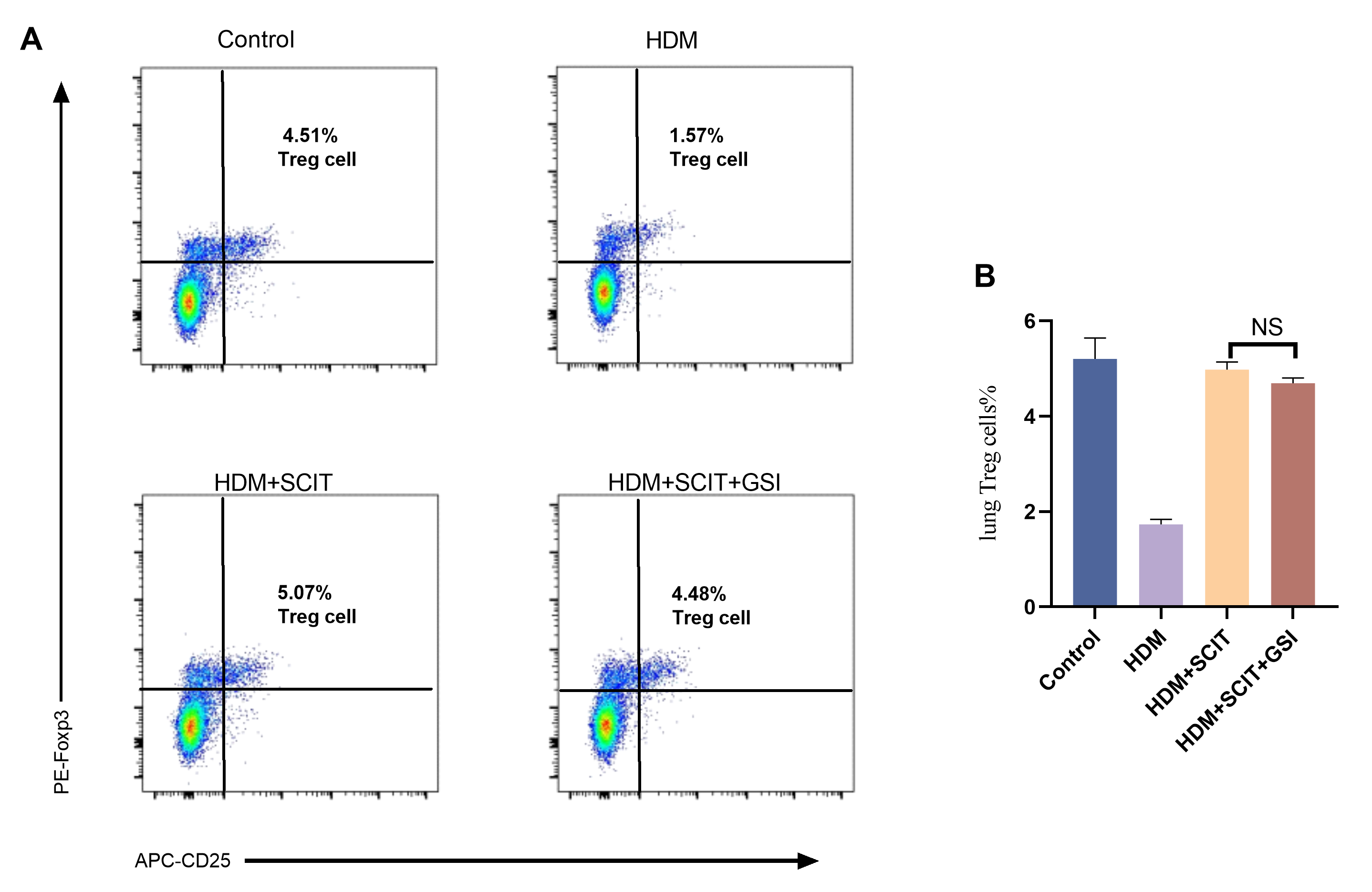


S7.(A) Flow cytometry strategy for gating of Treg cells (CD4^+^CD25^+^Foxp3^+^) in lung tissue. (B) Frequency of Treg cells in mouse lung tissue.


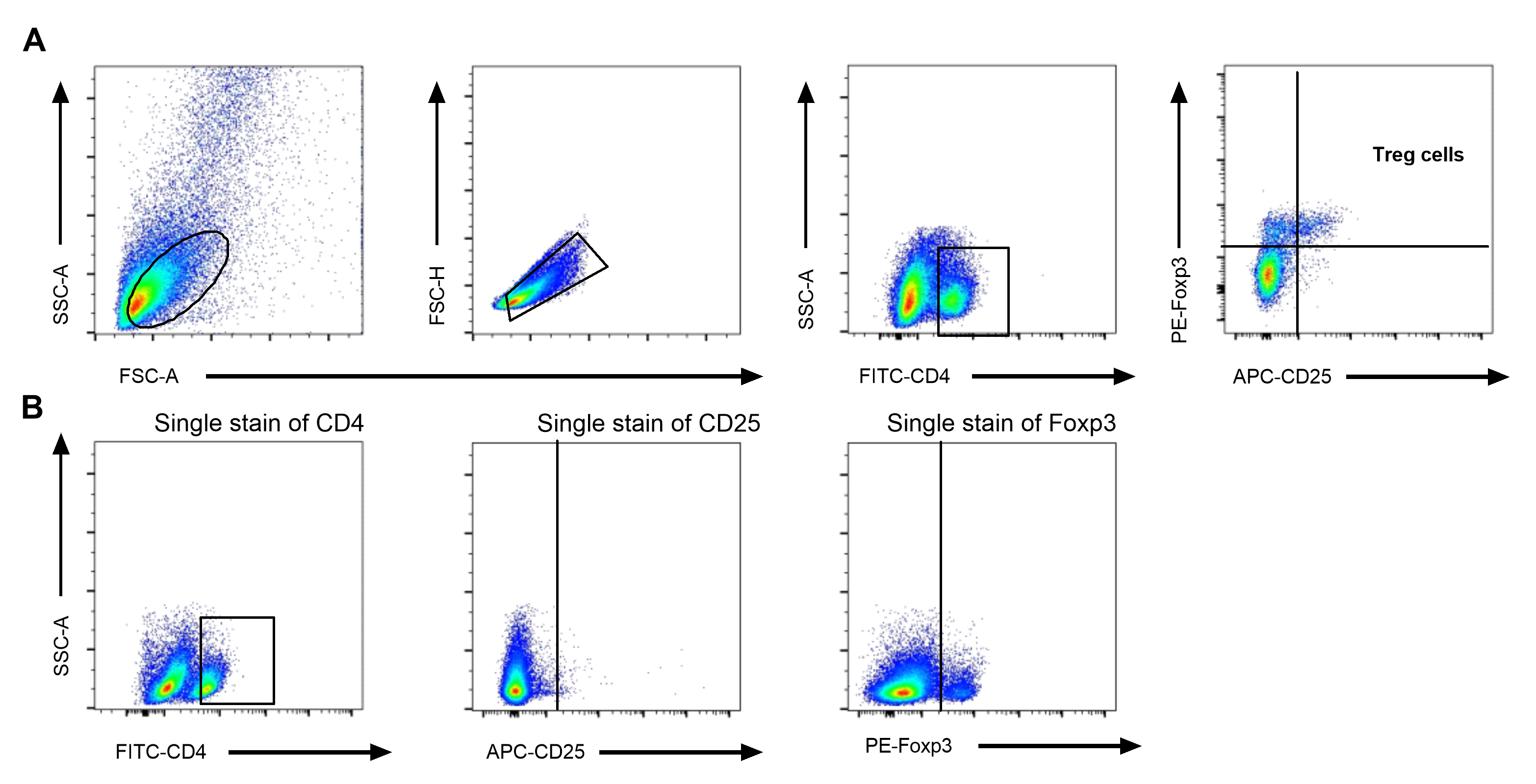


S8.Gating strategy for Treg cells. (A) Detailed sorting gating strategy diagram for Treg cells. (B) Single color staining for CD4, CD25, and Foxp3.

**
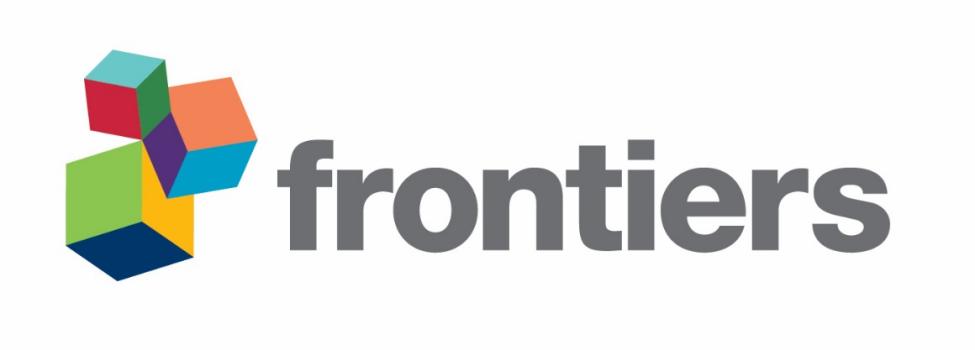
**
